# Phase Stability and Transitions in High-Entropy Alloys: Insights from Lattice Gas Models, Computational Simulations, and Experimental Validation

**DOI:** 10.3390/e27050464

**Published:** 2025-04-25

**Authors:** Łukasz Łach

**Affiliations:** AGH University of Krakow, Faculty of Metals Engineering and Industrial Computer Science, al. Mickiewicza 30, 30-059 Krakow, Poland; lach@agh.edu.pl

**Keywords:** high-entropy alloys, HEAs, phase stability, phase transitions, lattice gas models, computational modeling, mechanical properties

## Abstract

High-entropy alloys (HEAs) are a novel class of metallic materials composed of five or more principal elements in near-equimolar ratios. This unconventional composition leads to high configurational entropy, which promotes the formation of solid solution phases with enhanced mechanical properties, thermal stability, and corrosion resistance. Phase stability plays a critical role in determining their structural integrity and performance. This study provides a focused review of HEA phase transitions, emphasizing the role of lattice gas models in predicting phase behavior. By integrating statistical mechanics with thermodynamic principles, lattice gas models enable accurate modeling of atomic interactions, phase segregation, and order-disorder transformations. The combination of computational simulations (e.g., Monte Carlo, molecular dynamics) with experimental validation (e.g., XRD, TEM, APT) improves predictive accuracy. Furthermore, advances in data-driven methodologies facilitate high-throughput exploration of HEA compositions, accelerating the discovery of alloys with optimized phase stability and superior mechanical performance. Beyond structural applications, HEAs demonstrate potential in functional domains, such as catalysis, hydrogen storage, and energy technologies. This review brings together theoretical modeling—particularly lattice gas approaches—and experimental validation to form a unified understanding of phase behavior in high-entropy alloys. By highlighting the mechanisms behind phase transitions and their implications for material performance, this work aims to support the design and optimization of HEAs for real-world applications in aerospace, energy systems, and structural materials engineering.

## 1. Introduction

High-entropy alloys (HEAs) are metallic systems that contain five or more elements, each present in concentrations typically between 5% and 35 at.%. The term “high-entropy alloy” was first introduced in 2004 to describe alloys that defy conventional metallurgy by forming simple solid solutions despite their complex compositions. Unlike traditional alloys dominated by a single base element, HEAs derive their unique properties from high mixing entropy, which stabilizes disordered solid solution phases and suppresses the formation of brittle intermetallics, thereby enhancing mechanical strength, thermal stability, and corrosion resistance [1]. A key property influencing HEA performance is phase stability, which governs their structural and functional behavior. The intricate balance of entropic contributions, atomic interactions, and thermodynamic factors governs phase formation, stability, and transitions in HEAs, making them a subject of extensive research in materials science [2].

Phase transitions in HEAs significantly influence their mechanical and functional properties. These transitions, which include order-disorder transformations, precipitation hardening, and phase decomposition, dictate the alloy’s response to external stimuli such as temperature, pressure, and mechanical stress. Understanding these mechanisms is essential for tailoring HEAs to meet application-specific requirements. Some transitions enhance strength, while others improve ductility [3]. Due to thermodynamic and kinetic complexity, accurate prediction of phase behavior remains critical for advancing HEA design [4].

To investigate phase behavior in HEAs, researchers have increasingly used theoretical models, among which lattice gas models are notably effective. These models provide a statistical framework for analyzing atomic distribution, phase stability, and segregation in multi-component systems. By representing atoms on discrete lattice sites and considering their interactions, lattice gas models predict both equilibrium and non-equilibrium states. They effectively reveal the entropic and enthalpic drivers behind phase transitions [5]. External influences, such as temperature and composition gradients, are incorporated to yield insight into thermodynamic principles of HEAs [6].

Experimental validation complements modeling efforts in characterizing phase stability and transitions in HEAs. Advanced techniques like X-ray diffraction (XRD), transmission electron microscopy (TEM), and atom probe tomography (APT) provide critical data on atomic structure, phase composition, and microstructural evolution [7]. Coupling these methods with modeling improves phase prediction accuracy and guides alloy development. Furthermore, machine learning and data-driven approaches are increasingly being applied to analyze vast datasets and identify hidden correlations between alloy composition and phase behavior. These tools enable rapid exploration of compositions for targeted performance optimization [8].

Beyond structural roles, phase stability also influences HEAs’ functionality in catalysis, hydrogen storage, and thermoelectrics. Their ability to undergo reversible phase transformations without significant degradation makes them suitable candidates for energy-related technologies [9]. Thus, phase stability is crucial not only for mechanical robustness but also for enabling multifunctional capabilities.

This review focuses on the role of lattice gas models in analyzing and predicting phase transitions in HEAs. The discussion encompasses the fundamental principles governing HEA stability, including configurational entropy, lattice distortions, and atomic interactions, as well as the diverse phase transitions observed in these materials, such as order-disorder transformations, phase segregation, and spinodal decomposition. Additionally, the review evaluates the effectiveness of lattice gas models in capturing atomic-scale interactions and their integration with computational approaches—such as Monte Carlo simulations, molecular dynamics, and machine learning—to enhance predictive accuracy. It also discusses the synergy between advanced experimental techniques (XRD, TEM, APT) and modeling tools. By integrating theoretical and experimental advances, the review supports the design of HEAs with enhanced stability, mechanical strength, and functional versatility.

## 2. Fundamentals of Lattice Gas Models

Lattice gases are a theoretical framework that models fluids and gases as particles on a discrete lattice. This approach simplifies complex interactions, making it computationally feasible to simulate large systems. Lattice gas models are particularly useful in studying fluid dynamics, kinetic theory, and phase transitions. Rooted in statistical mechanics and cellular automata, they connect atomic-scale behavior to macroscopic properties.

**Figure 1:**
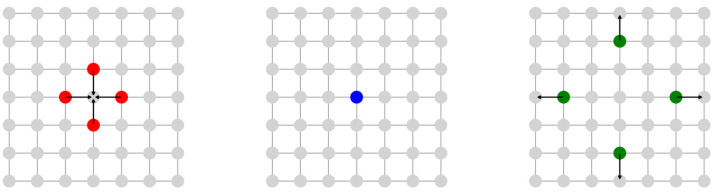
Stages of Multi-Particle Collision in a Lattice Gas Model.

LGCA models simulate fluid particles on a lattice using simple rules for collision and propagation. These models capture the microscopic behavior of fluids, leading to realistic macroscopic flow patterns. The Hardy, Pomeau, and de Pazzis model is a classic example of LGCA, demonstrating how simple rules can lead to complex fluid dynamics [10]. Figure 1 illustrates the three stages of a multi-particle collision in a lattice gas model. On the left, particles move toward the central node (red arrows). In the middle, they collide based on Lattice Gas Automata rules. On the right, they disperse outward, conserving momentum (green arrows). This visualization captures the microscopic dynamics of fluid motion in lattice-based simulations.

Lattice gas models are grounded in statistical mechanics, which provides a framework for understanding the equilibrium and transport properties of gases. These models are useful for studying phase transitions, diffusion, and other dynamic processes in solids and surfaces [11]. Lattice gases offer a simplified approach to solving kinetic theory of gases and fluid dynamics problems. They are used to simulate phenomena such as free expansion and compressible flow, providing insights into nonequilibrium systems [12,13]. Certain variants are linked to effective field theories, aiding in the analysis of fermionic behavior at low temperatures [14].

Lattice gas models are employed to simulate complex fluid dynamics problems, such as Rayleigh-Benard convection and Kelvin-Helmholtz instability. These simulations are often performed on parallel supercomputers, demonstrating the scalability and efficiency of lattice gas methods [15]. Lattice gas models have been applied to nuclear structure theory, providing a unified model that bridges strong and weak interaction approaches. They are also used to study the thermodynamics and kinetics of solids, including processes like electrical conductivity and aging [11,16].

While lattice gas models provide a powerful tool for simulating complex systems, they also have limitations. The discrete nature of the lattice can introduce artifacts that are not present in continuous systems, and the models may break down at low temperatures or high densities. Despite these challenges, lattice gases remain a valuable framework for exploring a wide range of physical phenomena, from fluid dynamics to quantum mechanics.

Classical and quantum lattice gas models differ significantly in computation, complexity, and application. Table 1 highlights the fundamental differences between classical and quantum lattice gas simulations.

Classical lattice gas models are traditionally used for simulating fluid dynamics and other physical phenomena, relying on cellular automata that scale linearly with system size and time steps. Quantum variants leverage unitary algorithms that may achieve exponential efficiency, especially for systems like Dirac particles coupled to gauge fields. This transition from classical to quantum models opens new avenues for simulating physical systems with greater accuracy and efficiency, albeit with challenges related to noise and state preparation in current quantum devices.

Classical LGCA scale linearly with system size and rely on local conserved quantities—factors that complicate their quantum adaptation [17,18]. Despite limitations, they remain widely used in fluid dynamics and complex system modeling [17].

Quantum lattice gas models can offer exponential advantages in space complexity, with quantum operations that are independent of system size [17]. Quantum algorithms for lattice gas automata demonstrate logarithmic complexity in terms of CXCX gates, suggesting potential for the efficient simulation of classical fluid dynamics on quantum devices [19]. Despite the impact of noise, accurate simulations are achievable on current noisy quantum devices, contingent on advancements in time step concatenation and state preparation [19]. Quantum extensions of classical models can encode complex algorithms like Simon’s algorithm, presenting challenges for efficient classical simulation [18].

Studies comparing quantum and classical spin models reveal that classical or semiclassical simulations can effectively analyze quantum many-body dynamics, even when the spin quantum number is small [20]. Quantum lattice models can exhibit unique structural properties, such as negative thermal expansion, which differ qualitatively from classical models [21].

Quantum lattice gas algorithms can efficiently model systems of Dirac particles interacting through gauge fields, preserving Lorentz invariance and avoiding the Fermi-sign problem [22]. Quantum lattice models can preserve continuous translation symmetry, offering new insights into emergent symmetries and potential laboratory demonstrations [23].

While quantum lattice gas models offer promising advantages over classical models, they also face challenges related to noise and the need for advanced quantum algorithms. The transition from classical to quantum models is not straightforward, as it involves overcoming significant technical hurdles to fully realize the potential benefits of quantum computation. Nonetheless, the development of quantum lattice gas models represents a significant step forward in the simulation of complex physical systems, with the potential to revolutionize fields such as fluid dynamics and quantum field theory.

Lattice gas models represent a significant methodological advancement in the domain of statistical thermodynamics, elucidating the microscopic foundations that underlie macroscopic thermodynamic characteristics. These models are particularly advantageous for investigating entropy’s significance in thermodynamic systems, as they facilitate the investigation of both configurational and translational entropy within a streamlined conceptual framework.

Lattice models are instrumental in merging configurational and translational entropy, which is crucial for a consistent thermodynamic model. By adopting the quantum volume as the volume of a lattice point, these models can derive refined equations of state, such as the Bragg-Williams equation, which generalizes the van der Waals equation of state [24]. The lattice gas models facilitate the derivation of well-known equations of state, including the ideal gas law and the van der Waals equation. They also incorporate temperature-dependent heat capacity, enriching conventional thermodynamic descriptions [24].

In active lattice gas models, entropy production is a continuous process at steady state. These models reveal complex phase diagrams and phase transitions, including first- and second-order nonequilibrium transitions, which are not typically observed in passive systems [25]. The study of entropy production in active lattice gases also highlights the occurrence of large deviations and intricate phase transitions, such as those found at a bias-induced tricritical point [25]. Thermodynamic integration helps compute configurational entropy in lattice-gas models. These calculations consider various factors such as anisotropy and energetic heterogeneity, providing a comprehensive view of entropy in different systems [26].

While lattice gas models offer significant insights into thermodynamic principles and entropy effects, they also present challenges and limitations. For instance, the simplifications inherent in these models may not fully capture the complexities of real-world systems, particularly those involving long-range interactions or non-equilibrium dynamics. Additionally, the assumptions made in defining lattice points and quantum volumes may not always align with experimental observations. Despite these challenges, lattice gas models remain a valuable tool for exploring fundamental thermodynamic concepts and continue to evolve with advancements in computational methods and theoretical frameworks.

Lattice gas models provide a powerful framework for understanding the complex interactions and behaviors in multi-component alloy systems. These models allow for the simulation of thermodynamic properties and phase behaviors by representing atoms or molecules on a lattice, where interactions are defined by specific rules. The analogy of lattice gas models to multi-component alloys is particularly useful in capturing the essence of atomic interactions and ordering phenomena in these systems. This approach is instrumental in studying the thermodynamic and kinetic properties of alloys, as well as their phase transitions and stability.

Lattice gas models can simulate multicomponent alloys with random interactions. By using distribution functions and integral equations within the pair approximation, it is possible to describe the interactions between different components in the alloy system. This approach helps in understanding the statistical mechanics of alloy systems with complex interactions [27]. In multi-component lattice gas systems, expressions for thermodynamic variables can be derived as functions of interaction parameters. These models can also quantify multi-point ordering, which is crucial for understanding the chemical ordering in alloys. The introduction of cluster order parameters allows for the systematic quantification of multi-point ordering motifs, which is essential for accurately describing the behavior of alloys with multiple components and sublattices [28,29]. Lattice gas models are also used to study the transport properties of alloys, such as mobility and interdiffusion. They align with Onsager transport theory to model kinetic behavior [30].

The cluster expansion method is a flexible lattice-based approach used to simulate the thermodynamic behavior of multicomponent alloys. This method allows for the decomposition of configurational energy into cluster interactions, providing a detailed understanding of the interactions among species in an alloy. The cluster decomposition is unique and invariant, offering a robust framework for parameter estimation and interpretation [31]. Variable-lattice models (VLM) refine traditional lattice models by incorporating atomic volume dependence on local environments. They better capture phase equilibria by addressing both short- and long-range atomic interactions [32].

While lattice gas models present a robust framework for the examination of multi-component alloys, they are not devoid of constraints. The intricacies inherent in real-world systems frequently necessitate the use of approximations, such as pair approximations, which may inadequately represent higher-order interactions. Furthermore, the foundational assumptions inherent in lattice models, including the imposition of fixed lattice structures and the employment of simplified interaction rules, may fail to encapsulate the dynamic characteristics of alloy systems. Notwithstanding these obstacles, lattice gas models continue to serve as an invaluable instrument for elucidating the fundamental properties and behaviors of multi-component alloys, thereby establishing a foundation for subsequent experimental and theoretical explorations.

## 3. Phase Transitions in High-Entropy Alloys

**Figure 2:**
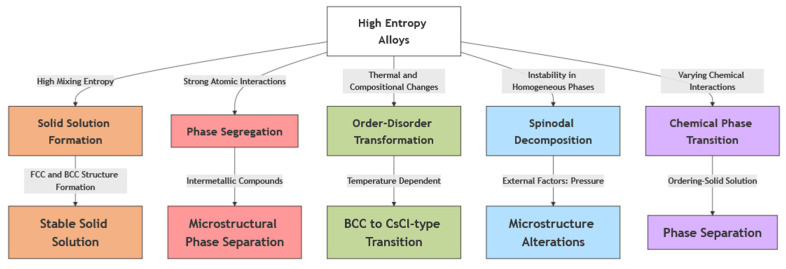
Phase Transitions in High-Entropy Alloys.

High-entropy alloys (HEAs) exhibit various phase transitions due to their multicomponent composition. These include solid-solution formation, phase segregation, and order-disorder transformations, which depend on atomic-scale interactions. Figure 2 provides a visual representation of these phase transitions, illustrating how mixing entropy, atomic interactions, and thermodynamic conditions drive structural changes in HEAs.

Owing to their high configurational entropy, HEAs typically favor the formation of disordered solid solutions such as face-centered cubic (FCC) or body-centered cubic (BCC) phases instead of ordered intermetallics [33,34]. However, depending on atomic size mismatch and enthalpic interactions, phase segregation, spinodal decomposition, and order-disorder transitions can also occur [34].

Despite the tendency to form solid solutions, HEAs can exhibit phase segregation when the interactions between certain elements are significantly different, leading to the formation of intermetallic compounds or elemental segregation [33]. Phase segregation can occur due to insufficient mixing entropy to overcome the differences in mutual solubility among the elements, resulting in microstructural phase separation [35].

Order-disorder transformations in HEAs involve transitions between ordered and disordered states, influenced by temperature and compositional changes [36]. These transformations are driven by changes in the internal energy and configurational entropy, which can be modeled using first-principles methods and statistical mechanics [36,37]. An example of such a transformation is the transition from a body-centered cubic (BCC) structure to a CsCl-type structure in the AlCrTiV HEA, which is temperature-dependent [37].

Spinodal decomposition is a type of phase transition where a homogeneous solid solution becomes unstable and decomposes into two or more phases with different compositions [38]. This process can be influenced by external factors such as pressure, which can significantly alter the microstructure and properties of HEAs [38].

HEAs can undergo chemical phase transitions, such as ordering-phase separation, where the tendency towards ordering and phase separation occurs at different temperature ranges [35]. These transitions involve changes in the chemical interactions between atoms, leading to structural transformations such as “ordering-solid solution” and “solid solution-phase separation” [35].

A comprehensive understanding of these transitions is essential to tailor HEAs for advanced engineering applications.

The influence of configurational entropy on phase stability in high-entropy alloys (HEAs) is a critical factor in determining their structural and functional properties. By mixing multiple principal elements, it favors disordered solid solutions over ordered phases. This effect, prominent in HEAs, distinguishes them from conventional alloys.

Configurational entropy stabilizes single-phase solid solutions, especially near equiatomic compositions. This is seen in FCC/BCC lattices via CALPHAD modeling [39]. The effect of configurational entropy is temperature-dependent. With higher entropy favoring the stability of disordered phases at elevated temperatures. In TiAl HEAs, vibrational and configurational entropy jointly influence stability across temperature [40]. In CrTaVW HEAs, configurational entropy affects the critical temperatures of phase transitions, as seen in the modulation of the TaCr2 Laves phase stability with varying V and W content [41].

While configurational entropy plays a crucial role, it must be balanced with mixing enthalpy to achieve phase stability. In high-entropy oxides, constituent chemistry also plays a decisive role [42]. In eutectic HEAs, phase stability is influenced by extreme pressures and temperatures, with configurational entropy contributing to the stability of phases like FCC under high-pressure conditions [43].

The predicted phase behaviors in HEAs, such as the CrTaVW system, have been validated experimentally, confirming the role of configurational entropy in phase stability [41]. In high-entropy solid-state electrolytes, configurational entropy enhances electrochemical stability, demonstrating its broader applicability beyond structural alloys [44].

Other factors, such as mixing enthalpy, atomic size differences, and electronic interactions, also play significant roles in phase stability. The interplay between these factors can lead to complex phase behaviors that require comprehensive thermodynamic and experimental analyses to fully understand. Additionally, the influence of configurational entropy may vary across different alloy systems and compositions, highlighting the need for tailored approaches in designing HEAs with desired properties.

Lattice distortions and atomic interactions also play a crucial role in the phase transitions of high-entropy alloys (HEAs). Lattice distortions, arising from the presence of atoms with different sizes and bonding characteristics, influence the stability, mechanical properties, and phase behavior of HEAs. Atomic interactions, including bonding and electronic structure, further modulate these effects, contributing to the unique phase transition behaviors observed in these materials.

Lattice distortions are inherent in HEAs due to the presence of multiple elements with varying atomic radii, leading to static and dynamic distortions that affect thermodynamic and mechanical properties [45]. Lattice distortions enhance mechanical properties such as yield strength and ductility by creating an uneven energy landscape that impedes dislocation movement, resulting in solid solution strengthening [46]. Lattice distortions stabilize the solid solution phase by increasing the effective temperature of the system, thus preventing phase separation or ordering [45]. This stabilization is crucial for maintaining the single-phase structure of HEAs [47].

The electronic structure and bonding characteristics, such as covalent and ionic bonds, influence the degree of lattice distortion and the resulting mechanical properties. For instance, (HfZrTaNbTi)C exhibits strong covalent bonds, enhancing stability [48]. Variations in interatomic bonding and partial charge distribution affect the mechanical properties and phase stability of HEAs. The total bond order density (TBOD) is a useful metric for understanding these interactions [49]. In some materials, electron-lattice coupling is essential for stabilizing certain phases, such as the insulating state in metal-insulator transitions, highlighting the importance of atomic interactions in phase transitions [50].

The inherent disorder and high configurational entropy in HEAs contribute to their unique phase transition behaviors, often stabilizing disordered single-phase structures [47]. Lattice distortions influence diffusion behavior, affecting phase transitions during processes like oxidation. For example, severe lattice distortion can lead to inhomogeneous diffusion and stress distribution, impacting phase stability [51].

The complexity of HEAs, with their multicomponent nature and high configurational entropy, presents challenges in fully understanding the interplay of these factors. Additionally, experimental and theoretical studies continue to explore the nuances of lattice distortions and atomic interactions, aiming to optimize the properties of HEAs for various applications.

Phase transitions in high-entropy alloys (HEAs) are complex phenomena influenced by various factors such as lattice distortions, pressure, and electronic configurations. Characterized by critical phenomena and universal scaling behaviors, these transitions provide insights into alloy stability, microstructural evolution, and mechanical performance.

Lattice distortions in HEAs are intrinsic due to the varying atomic radii, which facilitate the formation of single-phase solid solutions. These distortions can be static or dynamic, influenced by inter-atomic length disorder and temperature fluctuations, respectively. Recent studies propose a novel scaling law based on an effective temperature, unifying these effects across different alloy systems. This scaling law highlights the role of lattice distortion in enhancing the stability of solid solution alloys by effectively increasing the system’s temperature, which is particularly significant in HEAs [45].

Pressure plays a critical role in phase transitions of HEAs. For instance, in the MoxCrFeCoNi HEA system, a face-centered cubic (fcc) to hexagonal close-packed (hcp) transformation occurs under pressure due to electronic redistribution. The valence electron concentration is a key factor in regulating this transformation, with Mo doping encouraging the hcp phase. Such pressure-induced transformations offer strategic insights into alloy design for high-performance, load-bearing, or high-pressure environments [52].

Universal scaling laws, such as those described by the Kibble-Zurek mechanism, govern the density of topological defects during phase transitions. These laws apply to both equilibrium and non-equilibrium dynamic phase transitions, indicating that critical dynamics extend beyond traditional equilibrium criticality. This universality is crucial for understanding complex systems and their behavior under different conditions [53].

The electronic origin of phase transitions in HEAs is significant, as demonstrated by the pressure-induced transformations in AlCrMoSiTi HEAs. High pressure alters the microstructure and enhances corrosion resistance by changing the phase composition and volume fractions. Such electronic structure changes directly impact alloy stability and functionality, underscoring the role of electronic interactions in phase control [54].

The thermodynamic stability and phase transformations in HEAs are also influenced by topological factors. The distance between manifolds, as defined by fidelity and trace distance, can measure transformations between different topological phases. This approach provides a universal framework for understanding phase transitions in various topological systems, including HEAs [55].

**Figure 3:**
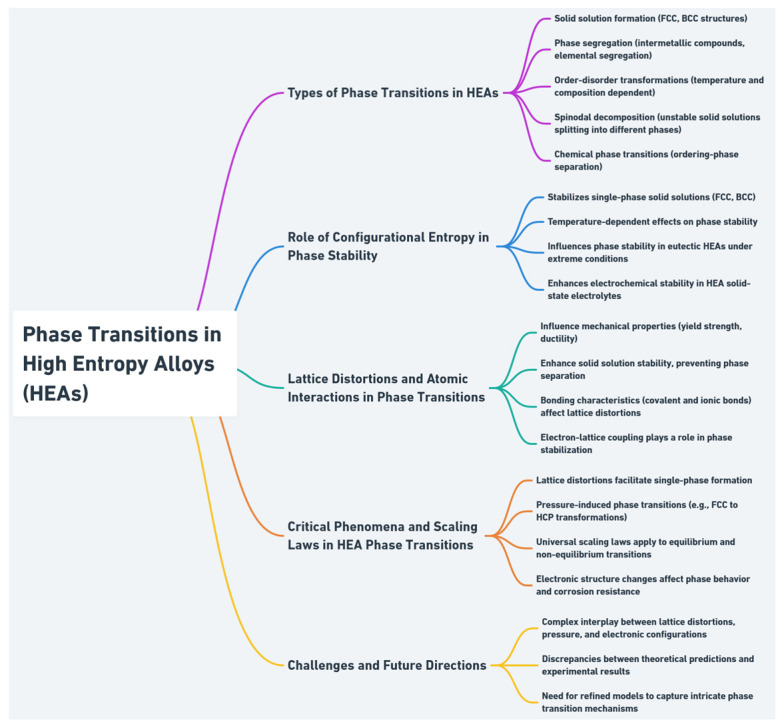
Summary of the key aspects related to the phase transitions in HEAs.

Despite recent advancements, fully capturing the interplay among lattice distortions, external fields (like pressure), and electronic effects remains a challenge. The development of universal scaling laws and critical phenomena provides a framework for further exploration, but discrepancies between theoretical predictions and experimental observations, such as those seen in AlCrFeMnMo HEAs, highlight the need for continued research and refinement of models [56]. Figure 3 presents a summary of the individual topics discussed in relation to the phase transitions in HEAs.

## 4. Application of Lattice Gas Models to HEA Phase Transitions

Lattice gas models, originally developed to simulate fluid dynamics and phase transitions in simple systems, have been adapted to model multi-component alloy systems like HEAs. These models use a discrete lattice framework to capture atomic interactions and configurational entropy, making them suitable for order-disorder transformations, phase separation, and spinodal decomposition in HEAs. The process of modeling phase stability and phase separation within high-entropy alloys (HEAs) presents a multifaceted challenge due to the extensive compositional landscape and the complex interactions among the constituent elements. A variety of methodologies, encompassing empirical models, machine learning algorithms, thermodynamic assessments, and computational simulations, are employed to predict phase stability and separation phenomena in HEAs. These methods aim to optimize the mechanical properties and stability of HEAs for practical applications.

The use of chromium equivalent (Creq) and nickel equivalent (Nieq) provides a comparative model to evaluate phase stability in HEAs. This approach classifies HEAs into different phases, such as FCC, BCC, and intermetallics, based on the calculated Creq and Nieq values, which correlate with valence electron concentration and melting temperature [57]. The Cahn-Hilliard equation, though initially formulated for binary systems, is used to model phase separation in HEAs. It provides a mathematical framework to simulate phase separation dynamics, such as spinodal decomposition, using various discretization methods [58].

Machine learning models, such as support vector machines and gradient boosting decision trees, are increasingly utilized for predicting phase formation and stability in HEAs. These models utilize datasets of HEAs to identify critical parameters like root mean square residual strain, which significantly influence phase stability [59].

CALPHAD (CALculation of PHAse Diagrams) modeling predicts HEA phase stability and transitions, with good experimental agreement [60,61]. Coupled with Virtual Crystal Approximation (VCA), it also estimates physical properties. For example, Co concentration in AlCoₓCrFeNi alloys was optimized to improve mechanical behavior [62].

A novel visualization technique using formation-energy and reaction-energy axes visualizes complex phase stability relationships in HEAs with more than four components. This method helps in understanding the transition from high-temperature stability to metastability [63]. In refractory HEAs, local disorder and short-range clustering significantly affect phase stability and strength, as revealed by ab initio simulations and advanced microscopy [64].

While these models provide valuable insights into phase stability and separation in HEAs, challenges remain due to the high dimensionality and kinetic effects. Integrating experimental data remains crucial. Continued methodological refinement is essential to enhance predictive capabilities.

Lattice gas models are well suited for modeling ordering–disordering transitions in multi-principal element alloys (MPEAs), capturing interactions arising from near-equiatomic compositions without a dominant solvent element. They model thermodynamic and structural features such as short-range order and lattice distortion that affect phase transitions. Table 2 outlines key boundary conditions in lattice gas models for HEA phase transitions, detailing their roles in diffusion, phase separation, and interfacial effects, ensuring realistic simulations.

Entropy plays a key role in disorder-to-order transitions in HEAs. Free energy calculations highlight the importance of anharmonic effects and chemical short-range order in transitions from a random solid solution to a more ordered phase [65]. SRO, predicted by quasi-chemical models, alters local structure and mechanical behavior in MPEAs. This is crucial for understanding phase stability and transitions in these alloys [66].

Machine learning models, including convolutional neural networks, predict phase structures in HEAs with high accuracy using large datasets and empirical descriptors [67,68]. Density-functional theory (DFT) and Monte Carlo simulations provide insight into phase equilibria and SRO, revealing effects on electronic and elastic behavior due to chemical ordering [66,69].

Lattice distortion and SRO affect HEA deformation and strengthening. Multiscale models combining atomistic simulations and crystal plasticity improve understanding of mechanical response [70]. Atomic electron tomography reveals 3D atomic positions and chemical order, confirming strain heterogeneity critical for property tuning [71].

Despite their utility, lattice gas models face limitations in capturing the full complexity of phase separation and interaction networks in HEAs [72]. The diversity of reactions and chemical heterogeneity complicates modeling, necessitating continued refinement of both experimental and computational approaches.

Lattice gas models effectively capture diffusion and kinetic processes during HEA phase transitions. By simulating particle dynamics on a lattice, they help predict critical behaviors that influence alloy performance.

Non-equilibrium models like the asymmetric exclusion process (TASEP) capture density-dependent phase regimes via repulsive interactions and flow boundaries [73]. In HEAs, they reveal how temperature, pressure, or particle flow affects phase evolution and mechanical response.

Models like the three-lane exclusion process simulate diffusion in HEAs, capturing fluctuation patterns and superdiffusive transport that influence phase kinetics and thermal behavior [74]. These models can simulate the movement of atoms or molecules within the alloy, offering a detailed view of how diffusion contributes to phase changes and the overall kinetics of the system.

The lattice Boltzmann method, combined with phase-field theory, models multiphase dynamics and density contrasts in HEAs [75]. It enables time-resolved simulations of phase development for alloy design.

Traditional lattice gas models may oversimplify by assuming discrete motion and nearest-neighbor interactions [76]. Their accuracy is also sensitive to boundary assumptions and dynamic rules.

Lattice gas models offer valuable insight into phase transitions and diffusion kinetics in HEAs. However, refining model assumptions and incorporating realistic interactions is essential for advancing predictive accuracy.

Lattice gas models, rooted in statistical mechanics, complement CALPHAD and ab initio methods by modeling thermodynamics through lattice-based interactions. Their integration improves prediction of phase behavior in HEAs.

Lattice gas models simulate multicomponent transitions by modeling interacting particles on a lattice. Tensor network approaches improve partition function calculation, enhancing prediction of critical behavior and chemical potential estimates [77,78].

CALPHAD remains a foundational method for modeling phase diagrams in HEAs, using Gibbs energy and mobility databases [79]. Lattice gas models add a microscopic view that complements CALPHAD’s macroscopic framework. Their integration improves the treatment of complex interactions in multicomponent systems [80].

First-principles methods (ab initio) reveal electronic structure and lattice stability, offering insight into the atomic-scale interactions driving HEA phase transitions [81,82]. Coupling these with lattice gas models bridges atomic-scale behavior with macroscopic phase evolution [83].

Integrating lattice gas, CALPHAD, and ab initio methods is challenged by the complexity of HEA composition spaces. However, coupling them with machine learning improves predictive accuracy [79]. Discrepancies between CALPHAD and DFT, such as lattice stability deviations, highlight the need for hybrid approaches [83].

**Figure 4:**
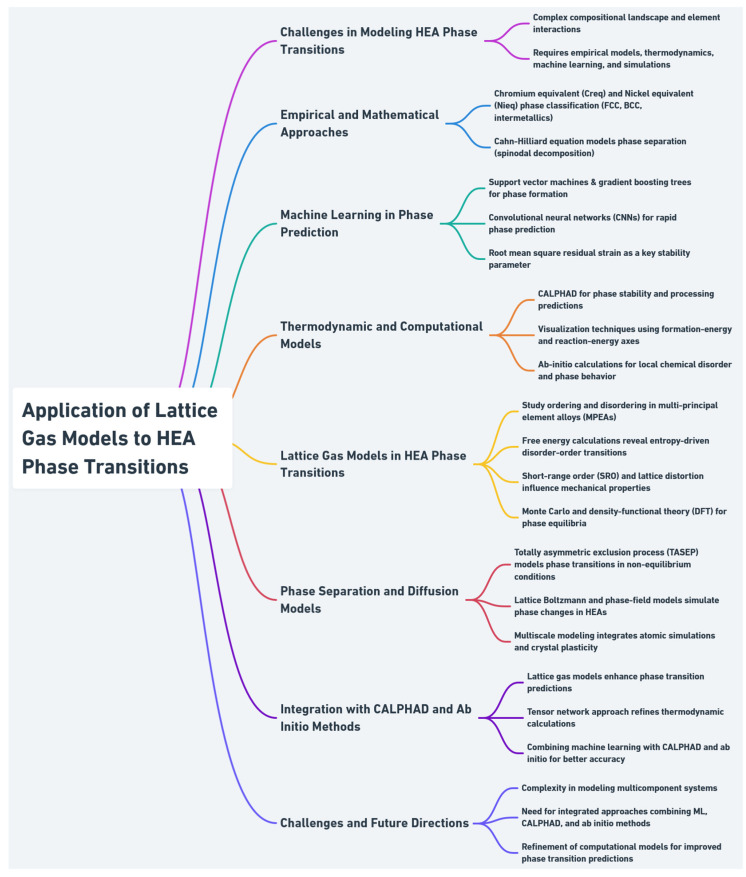
Overview of lattice gas model applications in HEA phase transitions.

Lattice gas models, while powerful, are limited by assumptions on potentials and geometry. Their integration with CALPHAD and ab initio tools is essential for accurately modeling complex phase behaviors in HEAs. Figure 4 illustrates a summary of findings on the application of lattice gas models to HEA phase transitions.

## 5. Computational and Simulation Techniques

This section reviews computational strategies for analyzing HEAs phase behavior. Monte Carlo, kinetic Monte Carlo, and molecular dynamics reveal atomic-scale interactions and kinetics. Machine learning methods leverage large datasets for phase prediction. Multi-scale frameworks bridge atomistic and continuum models, enabling comprehensive transition analysis.

### 5.1. Monte Carlo and Kinetic Monte Carlo Simulations in HEAs

Monte Carlo (MC) and kinetic Monte Carlo (KMC) methods simulate atomic-scale dynamics in HEAs. They are effective for modeling microstructural evolution, including chemical short-range order (CSRO) formation and defect phenomena such as helium bubble nucleation, which influence performance and longevity.

Monte Carlo methods use stochastic sampling to model thermodynamic states. The Metropolis algorithm finds equilibrium distributions in configuration space [84]. KMC extends this by incorporating real-time dynamics, enabling simulation of diffusion and radiation-induced microstructural changes [85]. KMC methods are based on the concept of variable time intervals between random events, which follow an exponential distribution [86].

KMC simulations reveal that sluggish diffusion is more pronounced in non-equiatomic HEAs [87]. Diffusion is governed by the potential energy landscape (PEL), shaped by chemical disorder and element-specific migration barriers [88]. Monte Carlo methods also evaluate thermal properties (e.g., conductivity, heat capacity), critical for high-temperature applications like nuclear reactors [89].

KMC simulations predict phase transitions in HEAs, such as FCC–BCC separation during annealing in AlCoCrFeMo_0.05_Ni_2_ [90]. They help evaluate metastability, addressing the limited thermodynamic data for multicomponent alloys [90].

KMC models construct time–temperature–CSRO diagrams for tailoring ordering kinetics in HEAs [91]. They also simulate helium trapping in nanostructured ferritic alloys, showing that Y-Ti-O nano-oxides prevent helium bubble formation, enhancing radiation tolerance [92]. This behavior is essential for improving radiation resistance and long-term performance.

MC–MD simulations analyze phase separation and microstructure evolution in HEAs. For example, FeTiTaVW exhibits BCC phase separation tied to thermal/mechanical behavior [89]. The simulations also help in understanding chemical short-range order (CSRO) in HEAs, which significantly affects their mechanical properties and irradiation resistance. For example, in HfNbTaTiZr HEAs, CSRO was found to influence the generation and accumulation of irradiation damage, enhancing the material’s resistance to dislocation generation [89].

Hybrid ML–MC frameworks improve HEA property prediction, such as migration barrier estimation [87]. Coupling Monte Carlo with first-principles simulations enhances understanding of atomic structures and phase transitions under thermal conditions [93,94].

Kinetic trapping in KMC occurs when systems remain in low-energy basins. This can be addressed using non-local jumps or absorbing Markov chains [85]. Accelerated KMC methods extend simulations to experimental time scales, useful in helium bubble studies [95]. Still, modeling HEA complexity requires multiscale and multiphysics integration to enhance prediction [96].

MC/KMC simulations are limited by the quality of input parameters (e.g., interatomic potentials, diffusion coefficients). Long-time or highly interactive systems can become computationally costly. Nonetheless, improvements in algorithms and computing power continue to expand their applicability in HEA research.

### 5.2. Molecular Dynamics Integration with Lattice Gas Models

Molecular dynamics (MD) simulations are essential for exploring atomic interactions in HEAs under varying temperatures, stresses, and compositions. They help reveal mechanical, structural, and phase transition behavior at the atomic scale.

MD simulations show that twin boundaries in CoCrFeNi-based HEAs block dislocation motion, improving ductility and strength. Precipitate morphology and interface structure further modulate defect evolution and mechanical response [97]. The mechanical properties of HfNbTaTiZr HEAs are influenced by the percentage of individual elements. For instance, adding Nb or Ta can enhance tensile strength and elastic modulus, demonstrating the importance of constituent composition in tailoring HEA properties [98]. Machine learning–driven MD, especially via graph neural networks, predicts properties by capturing local chemical ordering and atomic-scale structure–property relationships in medium-entropy alloys [99]. MD simulations have been used to study the plasticity and strength of equiatomic and non-equiatomic HfNbTaTiZr HEAs under uniaxial tensile loading. At high strain rates, both alloys behave similarly, but at lower rates, non-equiatomic compositions exhibit greater strength due to hcp atom formation, which enhances strength while reducing plasticity [100]. The mechanical properties of NbTiZrMoV HEAs are significantly influenced by temperature and strain rate. Higher temperatures reduce tensile strength and modulus, while faster strain rates enhance strength by restricting atomic rearrangements [101]. In HfNbTaTiZr HEAs, shock wave simulations show phase transformations from bcc to hcp and back, with twinning and dislocation activities contributing to spall behavior. Such reversible transformations under impact conditions are critical for high-resilience applications [102].

The FeNiCrCoCu HEA coatings demonstrate excellent wear resistance, though wear resistance declines at high temperatures due to increased lattice disorder [103]. The friction properties of HEA coatings are influenced by temperature-induced changes in lattice structure and dislocation density. Maintaining moderate temperatures helps retain lattice order and hardness, whereas high temperatures degrade these properties [103].

The addition of Cu in CoCrFeNi HEAs affects their mechanical properties and phase structures. Increasing Cu content initially enhances yield and tensile strength but eventually reduces them while consistently improving plasticity. This is attributed to changes in dislocation density and phase transitions from FCC to biphasic FCC structures [104]. The mechanical behavior of FeNiCrCoCu HEAs is also influenced by Twin Boundary Spacing (TBS). Smaller TBS sizes improve mechanical properties by facilitating dislocation migration and grain boundary diffusion, which are critical for enhancing strength and ductility [105].

MD simulations are limited by short time and length scales, requiring experimental validation. Integration with ML methods (e.g., graph neural networks) improves predictive power, especially for energy mapping and atomic interactions [99].

### 5.3. Machine Learning and Artificial Intelligence in Lattice Gas-Based Phase Prediction

Machine learning (ML) and AI are vital for navigating the high-dimensional composition space and nonlinear structure–property relations in HEAs. These tools accelerate property prediction and optimization for targeted alloy design.

ML models predict HEAs properties like yield strength and ductility. Evolutionary and Bi-Objective Genetic Programming (BioGP) methods have achieved up to 1795 ± 21 MPa yield strength and 31.45% ductility [106]. Bayesian-optimized regression models have also been applied to predict bulk modulus, demonstrating ML’s role in accelerated HEA discovery [107].

Soft computing models like artificial neural networks (ANN), k-nearest neighbor (kNN), and support vector machines (SVM) classify HEA phases (e.g., solid solution, amorphous, intermetallic), with kNN achieving 92% accuracy [108]. Advanced ML models (XGBoost, Random Forest) further enhance crystal structure prediction. To address class imbalance, Synthetic Minority Oversampling Technique (SMOTE) resampling has been applied [109].

ML combined with first-principles (DFT) modeling improves phase and property prediction. In Al-Co-Cr-Fe-Ni, this approach reveals correlations between composition, stability, and elasticity [110].

Explainable ML methods like Local Interpretable Model-agnostic Explanations (LIME) and Shapley Additive Explanations (SHAP) clarify how input features affect phase predictions in refractory HEAs [111]. In TiZrNbVAl design, feature selection was used to build accurate models for ductility prediction [112].

Despite their promise, ML models for HEAs are limited by data scarcity and quality. Experimental validation is essential for reliable predictions. Improved algorithms and curated datasets will enhance ML’s accuracy and applicability in materials design [113].

### 5.4. Multi-Scale Modeling Strategies for HEA Phase Transitions

Multiscale simulation frameworks are essential for capturing the mechanical behavior of HEAs across atomic to macroscopic scales. They integrate diverse computational tools to address structural and compositional complexity that governs key properties like strength, ductility, and toughness.

Crystal Plasticity Finite Element Method (CPFEM) integrates atomistic data into continuum models to predict mechanical properties like yield strength and stress–strain behavior. It captures strain localization due to lattice distortion and SRO [70]. Stochastic line tension models reduce computational load by estimating dislocation barriers, enabling efficient mechanical property prediction [114].

Multiscale frameworks link atomistic and continuum models to study crack tip plasticity and fracture resistance. They show how compositional complexity promotes dislocation emission and crack arrest [115]. These models also probe irradiation-induced microstructures, supporting the design of radiation-resistant HEAs for nuclear use [116].

Despite their power, multiscale simulations face limitations in accuracy, scalability, and computational cost. Integrating multiphysics models is essential to address these issues and enable practical HEA applications. Faster, scalable algorithms are needed to handle the vast HEA composition space and enhance predictive efficiency [117].

## 6. Experimental Validation and Real-World Applications

This section focuses on validating computational predictions using experimental phase diagrams of HEAs. It also highlights case studies where lattice gas models support HEA design. Real-world applications in aerospace, energy, and coatings underscore their industrial relevance.

### 6.1. Comparison of Lattice Gas Predictions with Experimental Phase Diagrams of HEAs

Comparing lattice gas predictions with experimental HEA phase diagrams requires assessing how statistical models capture phase behavior in multi-component systems. HEAs pose challenges due to their vast compositional space and complex interactions. Lattice gas models offer theoretical insights into phase transitions, but experimental validation remains essential.

Lattice gas models applied in statistical physics (e.g., biaxial nematics) simulate phase coexistence and criticality by accounting for isotropic interactions [118]. The two-dimensional lattice gas model, employing Monte Carlo simulations, offers a theoretical approach to predict phase diagrams, capturing phenomena like low-density solid phases and fluid phase transitions [119]. Though simplified, they often align qualitatively with experimental HEA phase trends. For example, experimental studies using X-ray diffraction (XRD) and atom probe tomography (APT) have confirmed phase separation patterns predicted by lattice gas simulations in CoCrFeNi and AlCoCrFeNi systems.

ML models predict HEA phases with high efficiency. A neural network trained on experimental data achieved 93.4% accuracy [120]. These models accelerate composition screening, reducing experimental demand. Advanced techniques like random forests and meta-learning further integrate empirical and computational data to improve prediction reliability [121,122].

Experimental validation is essential to confirm predictions from lattice gas and ML models. The CALPHAD method, for example, has been used to compare predicted phase formations with experimental observations, highlighting the influence of manufacturing processes on phase stability [123]. Combining simulations with experiments enhances model accuracy and reveals mechanisms behind phase transitions [96].

Despite their value, lattice gas and computational models often diverge from experiments due to effects like solute trapping and defect energetics. HEA complexity requires adaptable models that capture a wide range of interactions. Continued advances in computation and experimentation will improve prediction accuracy and accelerate alloy design.

### 6.2. Case Studies of Lattice Gas Applications in HEA Design and Optimization

Lattice gas models are used to design and optimize HEAs by simulating complex atomic behaviors. This section presents case studies showing how these models enhance alloy performance and structural properties.

Lattice gas models support thermodynamic analysis in CoNi-based high-entropy superalloys, enabling phase stability prediction and avoidance of precipitation or segregation during processing [124]. In automotive design, CAD and simulation tools guide HEA tailoring for lightweight, high-performance parts, supported by life cycle analysis [125]. The lattice gas models also optimize functionally graded lattice structures to balance stiffness and weight, critical for strength-to-weight efficiency [126].

Lattice gas models enable optimization of lattice architectures in additive manufacturing for applications like thermal conductivity enhancement and compliance reduction [127]. In turbine blade cooling, they balance thermal and mechanical performance, supporting the 3D-printed design of efficient, structurally robust topologies [128].

Machine learning frameworks have been employed to design HEAs with specific properties, such as high hardness and ductility. By constructing extensive datasets and employing feature selection and optimization algorithms, researchers have successfully predicted and validated new HEA compositions with enhanced mechanical properties [129]. Lattice gas simulations benefit from FPGA-based high-performance computing, accelerating complex fluid and phase dynamics [130]. The integration of lattice gas models with genetic algorithms provides a robust framework for optimization in continuous spaces. This approach enhances the exploration capabilities of genetic algorithms, leading to faster convergence and more comprehensive traversal of the solution space [131].

Lattice gas models are limited by interaction complexity and high computational cost in large HEA systems. However, advancements in computational techniques and additive manufacturing continue to expand the potential applications of lattice gas models in various engineering fields.

### 6.3. Industrial Applications

HEAs, composed of multiple principal elements, exhibit exceptional mechanical strength, corrosion resistance, and thermal stability. These attributes suit them for structural components, high-temperature applications, and protective coatings. However, production costs and processing challenges limit broader adoption.

HEAs maintain structural integrity under extreme conditions, making them suitable for aerospace components like turbine blades and engine parts [132]. Their strength-to-weight ratio, thermal stability, and corrosion resistance also enhance performance in lightweight automotive applications [125].

HEAs’ high configurational entropy enhances thermal stability, ideal for extreme-temperature industries like power generation and metallurgy [133]. High-entropy oxides (HEOs) offer further potential in heat-resistant coatings and components [134]. HEAs also show promise in solid-state hydrogen storage due to superior absorption and diffusion properties [135,136].

CoCrFeNi-based HEA coatings deliver excellent wear and corrosion resistance, suited for demanding industrial surfaces [137,138]. Techniques like plasma arc cladding enhance hardness and durability at low cost [139]. Cold spray deposition preserves microstructure by avoiding high-temperature transformations, maintaining coating density, and maintaining strength [138].

Additive manufacturing enables complex HEA components with tailored microstructures and minimal defects. AM enhances mechanical and corrosion performance, supporting applications in medical, aerospace, and nuclear sectors through design customization [140].

**Figure 5:**
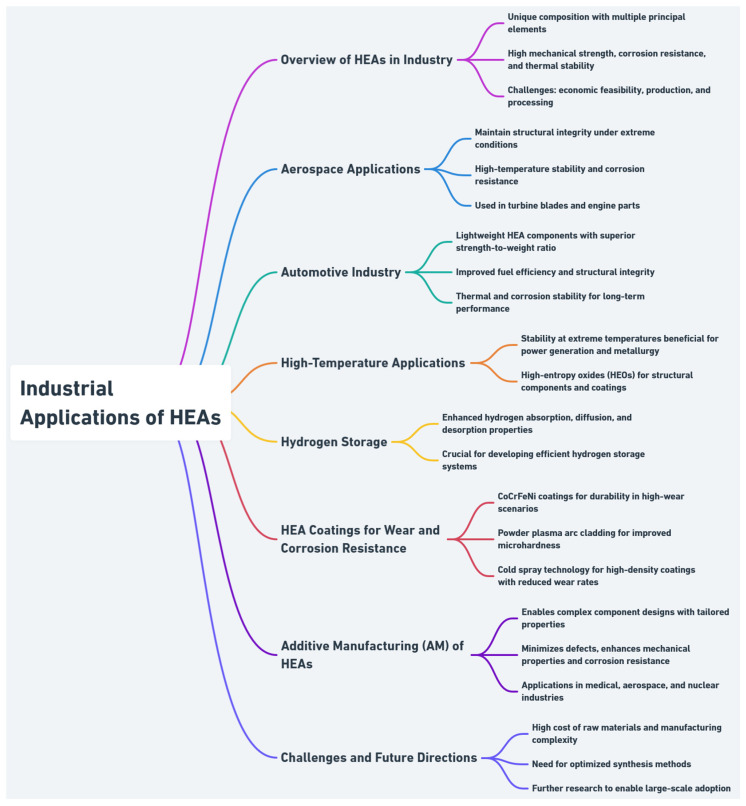
Overview of industrial applications of HEAs.

Despite their strengths, HEAs face challenges in cost-effective production and scalability. Raw material expenses and complex synthesis limit large-scale use. Further research is needed to optimize processing and deepen understanding of HEA behavior to enable broader industrial replacement of conventional alloys [132,133]. Figure 5 presents an overview of industrial applications of HEAs.

## 7. Challenges and Future Perspectives

This section addresses the limitations of lattice gas models in simulating complex multi-component HEAs. It explores hybrid strategies—like quantum lattice gases and DFT integration—to improve predictive accuracy. Future directions emphasize better simulation techniques and experimental validation to advance alloy design.

### 7.1. Limitations of Lattice Gas Models for Multi-Component Alloy Systems

Lattice gas models simulate multi-component alloys by capturing mesoscopic interactions. However, their accuracy is limited by challenges in thermodynamic consistency, boundary conditions, and representation of multi-species interactions.

Thermodynamic inconsistencies in lattice gas models can yield inaccurate phase behavior, especially in multi-phase HEAs [141,142]. These models may fail to capture branching effects and molecular interactions, impacting predictions of key properties like density and viscosity [143].

The lattice Boltzmann method (LBM), while effective for steady-state flows, struggles with modeling transient diffusion and velocity-viscosity accuracy in multi-component systems [144]. Percolation effects and mobility mismatches further limit predictive accuracy, complicating standard theories like Maxwell–Stefan [145].

Diffusion slip and other boundary conditions are difficult to implement in lattice gas models, affecting realism in interface-dominated systems [146]. Accurate results demand complex numerical setups, increasing computational cost [146].

Lattice gas models often support only low density ratios, making them inadequate for high-density-contrast systems [141].

Thermodynamic calculations via Monte Carlo methods are computationally intensive and algorithmically complex, reducing their accessibility for broader engineering use [147].

While lattice gas models provide a robust framework for multi-component alloy simulation, their limitations necessitate continued development. Improvements may include more advanced algorithms, refined boundary conditions, and better handling of complex interactions. Despite these challenges, lattice gas models remain a key tool in computational materials science, offering insights that are often inaccessible by other methods.

**Figure 6:**
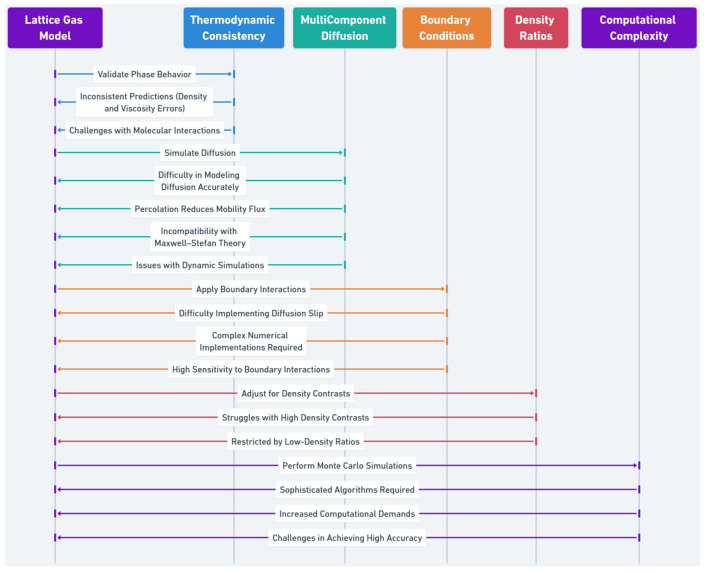
Limitations of lattice gas models in multi-component alloy simulations.

The sequence diagram (Figure 6) outlines key limitations of lattice gas models, including thermodynamic inconsistencies, diffusion challenges, boundary condition constraints, density ratio limitations, and computational complexity. It highlights issues such as inaccurate phase behavior, mobility flux reduction, numerical difficulties, and high computational demands, which impact the accuracy and efficiency of multi-component alloy simulations.

### 7.2. Opportunities for Hybrid Modeling Approaches

Hybrid modeling approaches, such as combining quantum lattice gases with DFT, offer enhanced accuracy in HEA simulations by integrating quantum and classical mechanics. These methods enable deeper insight into electronic structure but require high computational resources and careful parameter calibration.

Hybrid DFT models incorporating quantum mechanics better capture electronic interactions, particularly in strongly correlated systems [148]. The combination of DFT with machine learning techniques can accelerate the discovery and optimization of materials. For example, hybrid density functional theory (DFT) has been employed to facilitate the training of deep neural networks aimed at forecasting material characteristics, thereby significantly speeding up predictions compared to conventional methods [149]. Hybrid quantum-classical models can tackle high-dimensional problems that are computationally intractable using only classical methods. These models leverage quantum computing’s potential to solve complex optimization problems, which is crucial for simulating large-scale systems like HEAs [150].

Despite their potential, hybrid simulations often impose substantial computational costs. Efficient execution relies on techniques such as GPU acceleration and seminumerical integration [151]. Accurate parameterization is equally important and often necessitates data-driven techniques to approximate unknown correlations in mechanistic models [152,153]. Furthermore, hybrid models—especially those blending quantum and molecular mechanics—must carefully treat system–environment interactions, including electrostatic and dispersion forces, which strongly influence thermodynamic predictions [154].

As quantum computing progresses, hybrid quantum-classical models will increasingly solve problems previously out of reach, enabling advances in complex materials simulations [150]. Multiscale models, such as DFT with classical embedding, improve predictions across length scales [154]. The future of hybrid modeling lies in collaborative efforts across disciplines to develop standardized methods and share data. This will facilitate the broader application of hybrid models in various fields, including material science and structural biology [155].

Though promising for HEAs and complex systems, hybrid methods require significant computational resources, precise parameterization, and accurate treatment of system–environment interactions. With continued technical progress, they are poised to become central to materials science.

### 7.3. Future Research Directions in HEA Phase Transformation Modeling

Future advancements in high-entropy alloy (HEA) phase transformation modeling will be driven by the integration of advanced computational methods and targeted experimental validation. Emphasis is placed on the synergy between machine learning, phase-field modeling, and atomic-scale simulations to navigate the inherent complexity of HEAs.

Machine learning (ML) algorithms, such as XGBoost and random forest, offer strong predictive capabilities for HEA phase transformations, particularly when trained on data from electron backscatter diffraction (EBSD) and crystal plasticity finite element modeling (CPFEM) [156,157]. Embedded atom method (EAM)-based potentials can support molecular dynamics (MD) simulations by capturing martensitic mechanisms and cohesive energy effects [158]. Enhancing these models could improve prediction precision and broaden their applicability across a wider range of HEA systems. Advanced deep learning methodologies, including regularized deep neural networks and generative adversarial networks, present opportunities for the optimization of phase prediction models and the generation of supplementary data samples that mitigate data scarcity issues [159].

Phase-field modeling remains a key method for capturing microstructural evolution and phase transformation pathways in multiphase HEAs, particularly regarding equilibrium volume fractions, free energy landscapes, and elastic mismatches [160]. Complementary atomic-scale simulations—such as Monte Carlo and molecular dynamics—offer detailed insights into nucleation processes and the influence of alloy composition and thermal treatment on phase stability [56,161].

EBSD and TEM are essential for validating computational models and tracking microstructural changes during HEA phase transformations [162]. Data-driven approaches can benefit from data augmentation strategies to address class imbalances in datasets, thereby improving the robustness and accuracy of ML models in predicting HEA phases [163]. It is important to study the kinetics of strain-induced phase transformations and the effects of lattice distortions on phase stability [164]. Investigating bidirectional transformations and reverse transformations in HEAs can offer a deeper understanding of the deformation mechanisms and guide the design of alloys with enhanced mechanical properties [165].

Future research should prioritize the design of HEAs with application-specific microstructures by linking phase transformation pathways to alloy composition and processing conditions [160]. Key parameters—such as mixing enthalpy and valence electron concentration—remain critical for guiding phase stability and performance optimization [156,159]. The exploration of new HEA systems, including high-entropy shape-memory alloys (HESMAs), can expand the range of functional properties available for engineering applications. Research should focus on understanding the martensitic phase transformation and functional properties of these alloys [166]. Soft computing techniques, such as artificial neural networks and support vector machines, can be optimized to enhance phase prediction accuracy, facilitating the discovery of novel HEA phases [108].

Advancing phase transformation modeling in HEAs requires more than computational and machine learning integration— it demands consistent experimental validation and cross-disciplinary coordination. The complexity of HEA systems, defined by their vast compositional and structural variability, underscores the need for cohesive frameworks that bridge theory, simulation, and experiment.

## 8. Conclusions

High-entropy alloys (HEAs) are an emerging class of materials composed of multiple principal elements in near-equimolar ratios. This distinctive composition imparts exceptional mechanical strength, thermal stability, and resistance to wear and corrosion. A critical factor influencing these properties is phase stability, which determines the structural integrity and functional adaptability of HEAs under varying conditions. The intricate balance between entropic and enthalpic contributions governs phase formation and transitions, making phase stability a central focus in HEA research. Understanding these phase transformations is essential for tailoring HEA properties to meet the demands of advanced engineering applications.

This review highlights the role of lattice gas models as a robust statistical framework for modeling phase behavior in HEAs, particularly in relation to atomic interactions, phase segregation, and ordering phenomena. These models complement experimental methods such as X-ray diffraction (XRD), transmission electron microscopy (TEM), and atom probe tomography (APT), which provide atomic-level insights into structure and microstructural evolution. Integrating computational modeling with experimental validation significantly improves the predictive accuracy of phase behavior and accelerates the development of HEAs with tailored properties.

Recent advances in machine learning and data-driven methodologies have further enhanced phase behavior analysis, enabling high-throughput screening of alloy compositions. These tools facilitate the identification of thermodynamically stable configurations and enable precise prediction of phase transitions. The combination of lattice gas modeling, computational techniques, and experimental data deepens the understanding of phase transformations in HEAs and informs the design of alloys with enhanced mechanical and functional performance.

The synergy between modeling and experimental approaches is critical to advancing HEA research. By synthesizing recent developments, this review supports the optimization of HEAs for demanding applications in aerospace, automotive, and energy sectors. The insights gained provide a foundation for designing next-generation HEAs with superior phase stability, mechanical resilience, and multifunctional. As HEAs continue to evolve, these advances will drive broader industrial adoption and open new frontiers in materials innovation.

## Figures and Tables

**Table 1:** Comparison of Classical vs. Quantum Lattice Gas Models.

Aspect	Classical Lattice Gas	Quantum Lattice Gas
Computational Complexity	O(N)—Linear Complexity	O(log N)—Logarithmic Complexity
Scaling Behavior	Scales linearly with system size	Scales logarithmically with system size
Efficiency	Computationally expensive for large systems	Exponential efficiency gain for large systems
Memory Usage	High, increases with system size	Low, due to quantum parallelism
Simulation Type	Cellular automata based on rule-based updates	Quantum algorithms leveraging entanglement
Parallelism	Limited paralel processing	Inherent quantum parallelism
Key Advantage	Simple, well-studied for fluid dynamics	Massive speedup for large simulations
Limitation	Limited scalability; high computational cost	Requires advanced quantum hardware; noise-sensitive
Real-World Applications	Computational fluid dynamics, turbulence modeling	Quantum fluid dynamics, quantum field theory, Dirac equation simulations
Energy Efficiency	High energy consumption due to large computations	Lower energy consumption due to computational efficiency
Experimental Implementation	Easily implemented on classical supercomputers	Requires quantum computers with high coherence times
Error Sensitivity	Low; numerical precision issues in high-resolution simulations	High; noise and decoherence affect computations
Hardware Requirements	Traditional CPU/GPU clusters, supercomputers	Quantum processors (e.g., superconducting qubits, trapped ions)

**Table 2:** Boundary Conditions in Lattice Gas Models for HEA Phase Transition Modeling.

Boundary Condition	Description	Applications in HEA Modeling	Effect on Simulation
Periodic Boundary Conditions (PBCs)	The system repeats itself at the boundaries, simulating an infinite medium.	Spinodal decomposition, Ordering/disordering transitions, KMC/MD/MC simulations.	Eliminates artificial boundaries, mimicking an infinite system. Prevents finite-size effects, ensuring realistic phase behavior.
Fixed/Dirichlet Boundary Conditions	The values at the boundary are fixed, representing external constraints.	Ordering/disordering transitions, Interfaces/surfaces, Stress-induced phase transitions.	Models fixed temperature, concentration, or stress conditions. Simulates experimental constraints like boundary layers in coatings.
Reflective/Neumann Boundary Conditions	No flux condition at the boundary, ensuring no particle or energy flow across it.	Spinodal decomposition, KMC simulations, Defect evolution.	Ensures no loss of mass or energy. Useful for modeling confined HEA nanostructures. Prevents artificial interactions with an external environment.
Open Boundary Conditions (OBCs)	Allows mass or energy exchange with the surroundings.	Spinodal decomposition, KMC simulations, Nonequilibrium HEA systems.	Models external interactions like evaporation, adsorption, or material influx. Captures real-world experimental conditions better.
Mixed (Robin) Boundary Conditions	A combination of Dirichlet and Neumann conditions, balancing function values and their derivatives.	Interfaces and surfaces, Gradient-driven transitions.	Controls interactions at interfaces. Allows gradual diffusion between regions instead of sharp discontinuities.
Absorbing Boundary Conditions	Particles or energy reaching the boundary are removed from the system.	Defect migration, Interfacial reactions, Surface evaporation models.	Models processes where materials are lost, such as oxidation or desorption. Simulates degradation effects in HEA coatings.

## Data Availability

No new data were created or analyzed in this study. Data sharing is not applicable to this article.
